# Phytobezoar-Induced Jejunal Obstruction in an Elderly Male With Multiple Comorbidities

**DOI:** 10.7759/cureus.105285

**Published:** 2026-03-15

**Authors:** Saima Nawab, Muhammad Ali Shah, Rajesh Nambiar, Ahmer A Longi, George Alexander

**Affiliations:** 1 Internal Medicine, Mediclinic Welcare Hospital, Dubai, ARE; 2 General Surgery, Mediclinic Welcare Hospital, Dubai, ARE; 3 Gastroenterology and Hepatology, Mediclinic Welcare Hospital, Dubai, ARE

**Keywords:** gastrointestinal bezoar, intestinal obstruction, jejunal obstruction, phytobezoar, small bowel obstruction

## Abstract

Phytobezoars are rare causes of small bowel obstruction, most often affecting older adults with multiple comorbidities. Their diagnosis is frequently delayed because the presenting symptoms are vague and nonspecific. We report the case of an 84-year-old male with several chronic conditions who presented with persistent hiccups, vomiting, and reduced oral intake. Initial conservative management was undertaken; however, subsequent imaging, including CT scans and barium studies, demonstrated a complete blockage in the proximal jejunum. Exploratory laparotomy revealed a large intraluminal mass, which was successfully removed through transverse enterotomy. The patient required short-term vasopressor support after surgery and was discharged without complications on postoperative day 7. This case highlights that the early use of multimodal imaging and prompt surgical intervention is essential for accurate diagnosis, effective management, and uneventful postoperative recovery.

## Introduction

Bezoars are aggregations of indigestible material accounting for 0.4-4% of all mechanical bowel obstructions [1]. They are categorized based on composition into phytobezoars (plant material), trichobezoars (hair), pharmacobezoars (medications), and lactobezoars (milk products) [2]. A large bezoar size and elevated CT attenuation values may indicate the need for surgical intervention. Surgery remains a necessary and effective option when conservative management fails [3].

Recent studies note that phytobezoar-related small bowel obstruction is uncommon yet clinically significant, particularly in older or high-risk patients. Reports suggest that phytobezoars may form even in individuals without prior gastric surgery, commonly linked to high-fiber diets, poor mastication, and reduced gut motility. For example, phytobezoar-related obstruction can occur in the elderly [4] or dialysis patients with low activity levels and high consumption of certain plant foods [5].

A rare instance of phytobezoar linked with an intraluminal polyp highlights the need for timely diagnosis to avoid complications [6]. Phytobezoars, often resulting from excessive intake of high-fiber foods, such as cabbage, corn, grape skins, and seeds, may cause symptoms ranging from abdominal discomfort to intestinal obstruction or perforation, though they can remain asymptomatic [7]. This case underscores the importance of early recognition of bezoars, especially in elderly patients with cognitive impairment or multiple comorbidities, where symptoms may be subtle or atypical.

## Case presentation

An 84-year-old male with a background of ischemic heart disease, coronary artery bypass graft, heart failure (ejection fraction 30%), Alzheimer’s dementia, type 2 diabetes mellitus, benign prostatic hyperplasia, and bipolar disorder was brought to the emergency department in May 2025 with persistent hiccups, non-bilious vomiting, and decreased oral intake lasting over 24 hours. Despite ongoing passage of stools, the family reported reduced activity and fluctuating alertness.

The initial evaluation revealed stable vital signs and mild hypoactivity. Troponin-T was mildly elevated at 36 ng/L. Chest X-ray showed a chronic left-sided pleural effusion. A nasogastric tube was inserted, yielding approximately 1 L of bile-stained fluid. Despite initiation of proton pump inhibitors (PPIs) and prokinetics, the patient’s condition worsened, with episodes of coffee-ground emesis.

**Figure 1:**
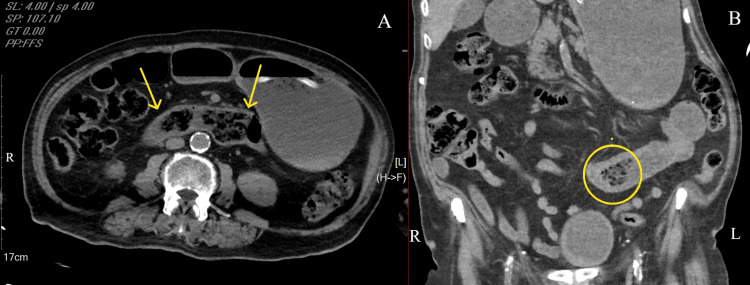
CT scan of the abdomen showing a phytobezoar at different levels of the jejunum. (A) Axial contrast-enhanced CT showing gastric distension and a mottled intraluminal mass in the proximal jejunum (phytobezoar indicated by yellow arrows). (B) Coronal CT image demonstrating a circumscribed jejunal phytobezoar (yellow circle) with mottled gas density resulting in upstream small bowel dilatation.

An upper GI endoscopy revealed retained food and bile in the stomach but no obstructive lesions or ulcers. Abdominal CT showed massive gastric distension with dilated proximal small bowel loops, raising suspicion of high-grade obstruction (Figure 1A, 1B). A barium follow-through was performed, confirming a complete luminal obstruction in the proximal jejunum.

**Figure 2:**
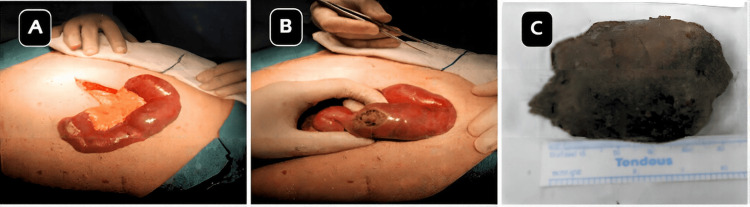
(A) Intraoperative image before opening the jejunum. (B) Intraoperative image after opening the jejunum. (C) Image with measurements of the phytobezoar extracted postoperatively.

Following multidisciplinary consultation, the patient underwent exploratory laparotomy. A large, firm intraluminal mass measuring approximately 6 × 5 cm was identified 45 cm distal to the duodenojejunal flexure. A transverse enterotomy was performed, and a phytobezoar composed of undigested plant fibers was extracted (Figure 2A-2C).

Postoperatively, the patient required transient vasopressor support due to intraoperative hypotension but stabilized within 24 hours. He was successfully extubated the following day, resumed oral intake within 48 hours, and had normal bowel movements. He was discharged home on postoperative day 7 in stable condition.

At one-week post-discharge follow-up, the patient was clinically stable with no new complaints. Surgical review showed a well-healing wound, and cardiology follow-up led to resumption of guideline-directed medical therapy.

## Discussion

Phytobezoar-induced small bowel obstruction is an uncommon but important cause of acute abdomen, particularly in elderly patients with multiple comorbidities. High-fiber food ingestion, even without prior gastric surgery, can precipitate bezoar formation. Gastric bezoars may be treated conservatively, endoscopically, or surgically. Most patients presenting with gastric phytobezoars can be successfully treated by consuming carbonated beverages in conjunction with endoscopic therapy [8]. Double-balloon enteroscopy, when combined with sequential laxative therapy, offers an effective treatment option for managing breakable phytobezoars. However, small bowel obstructions may occur due to the migration of a fragmented bezoar, necessitating surgical intervention. Laparoscopic removal offers favorable postoperative outcomes but carries risks of intra-abdominal spillage and longer operative time [9].

In the present case, multimodal imaging with CT and barium follow-through was pivotal in localizing and characterizing the proximal jejunal obstruction caused by a phytobezoar. Studies reinforce the importance of structured, multimodal diagnostic strategies in improving early detection and guiding effective management of bezoar-induced obstructions. Kaba et al. [10] emphasized that timely application of targeted imaging, such as whole abdominal ultrasonography, and adherence to structured diagnostic algorithms can reduce diagnostic delays, particularly in rare bezoar-related obstructions. Similarly, Wang et al. [11] demonstrated that integrating quantitative CT analysis with the AGESS-SBO scoring system enhances diagnostic accuracy in suspected phytobezoar-induced small bowel obstruction.

In the present case, conservative measures such as nasogastric decompression, PPIs, and prokinetics were insufficient due to the high-grade nature of the obstruction, necessitating surgical intervention. Patel et al. [12] highlighted that complex gastrointestinal motility disorders, as in cystic fibrosis, may not fully respond to conservative strategies alone and require multidisciplinary, sometimes invasive, management to restore function.

In our case, exploratory laparotomy with transverse enterotomy and bezoar extraction led to rapid recovery and restoration of bowel function. Claro et al. [13] highlighted that a high index of suspicion and early radiological evaluation are critical for timely diagnosis and effective management of bezoar-induced obstruction, given its nonspecific clinical presentation. Furthermore, the multidisciplinary considerations discussed in Bellezzo et al. [14] resonate with our approach, as patients with complex comorbidities or multisystem involvement benefit from coordinated care to address gastrointestinal dysfunction alongside other health concerns, ultimately improving postoperative recovery and quality of life.

Although the incidence of phytobezoar-induced small bowel obstruction is low, it should be suspected in patients with persistent vomiting and gastric distension, particularly in the absence of malignancy, strictures, or other common etiologies. A high index of suspicion is warranted when multimodal imaging identifies an intraluminal mass with characteristics suggestive of a bezoar. Management usually consists of nasogastric decompression, electrolyte balance, and surgical extraction in cases of complete or high-grade obstruction [4,15]. Prompt intervention coupled with supportive care generally results in complete recovery within days to weeks, whereas delayed diagnosis significantly heightens the risk of severe complications, including perforation and peritonitis [16,17].

Phytobezoars are primarily composed of indigestible plant material such as cellulose, lignin, and tannins, often found in high-fiber foods like persimmons, celery, and citrus fruits [18]. Risk factors for their formation include gastroparesis (commonly seen in diabetics), previous gastric surgery, inadequate mastication (especially in denture users), and prolonged immobility or cognitive decline [19,20].

Although phytobezoars typically form in the stomach, they can migrate distally, leading to small bowel obstruction, a less common but more serious manifestation [21]. In patients with dementia, as in this case, the diagnosis may be delayed due to nonspecific or masked symptoms such as lethargy, hiccups, or subtle changes in oral intake. Routine abdominal X-rays often lack specificity, and endoscopy may not visualize beyond the stomach. Therefore, CT imaging and contrast studies, such as a barium meal, remain valuable diagnostic tools [22]. When conservative measures such as enzyme therapy or endoscopic removal are not feasible or fail, surgical intervention becomes necessary. In this case, surgical enterotomy allowed complete resolution of the obstruction, with excellent postoperative recovery.

## Conclusions

Although rare, phytobezoar-induced small bowel obstruction poses a considerable diagnostic and therapeutic challenge, particularly in elderly individuals with multiple comorbidities and impaired gastrointestinal motility. Because symptoms such as vomiting, abdominal distension, and reduced oral intake are often nonspecific, a comprehensive clinical evaluation supported by early imaging is critical for determining the underlying cause of obstruction and preventing serious complications, such as perforation or ischemia. Effective multidisciplinary collaboration among physicians, radiologists, and surgeons is crucial for prompt decision-making and reducing perioperative risks. Preventive measures, including nutritional guidance and regular monitoring of individuals at higher risk, play a vital role in minimizing recurrence and promoting better long-term health outcomes.
